# WRN-targeted therapy using inhibitors NSC 19630 and NSC 617145 induce apoptosis in HTLV-1-transformed adult T-cell leukemia cells

**DOI:** 10.1186/s13045-016-0352-4

**Published:** 2016-11-09

**Authors:** R. Moles, X. T. Bai, H. Chaib-Mezrag, C. Nicot

**Affiliations:** 1 Department of Pathology and Laboratory Medicine, Center for Viral Oncology, University of Kansas Medical Center, 3901 Rainbow Boulevard, Kansas City, KS 66160 USA; 2 Department of Pathology and Laboratory Medicine, Center for Viral Oncology, KU Cancer Center, University of Kansas Medical Center, 3901 Rainbow Blvd, Kansas City, KS 66160 USA

**Keywords:** Human T-cell leukemia virus type 1 (HTLV-1), Adult T-cell leukemia/lymphoma (ATLL), WRN helicase, Cycle arrest, Apoptosis

## Abstract

**Background:**

Human T-cell leukemia virus type 1 (HTLV-1) infection is associated with adult T-cell leukemia/lymphoma (ATLL), a lymphoproliferative malignancy with a dismal prognosis and limited therapeutic options. Recent evidence shows that HTLV-1-transformed cells present defects in both DNA replication and DNA repair, suggesting that these cells might be particularly sensitive to treatment with a small helicase inhibitor. Because the “Werner syndrome ATP-dependent helicase” encoded by the WRN gene plays important roles in both cellular proliferation and DNA repair, we hypothesized that inhibition of WRN activity could be used as a new strategy to target ATLL cells.

**Methods:**

Our analysis demonstrates an apoptotic effect induced by the WRN helicase inhibitor in HTLV-1-transformed cells in vitro and ATL-derived cell lines. Inhibition of cellular proliferation and induction of apoptosis were demonstrated with cell cycle analysis, XTT proliferation assay, clonogenic assay, annexin V staining, and measurement of mitochondrial transmembrane potential.

**Results:**

Targeted inhibition of the WRN helicase induced cell cycle arrest and apoptosis in HTLV-1-transformed leukemia cells. Treatment with NSC 19630 (WRN inhibitor) induces S-phase cell cycle arrest, disruption of the mitochondrial membrane potential, and decreased expression of anti-apoptotic factor Bcl-2. These events were associated with activation of caspase-3-dependent apoptosis in ATL cells. We identified some ATL cells, ATL-55T and LMY1, less sensitive to NSC 19630 but sensitive to another WRN inhibitor, NSC 617145.

**Conclusions:**

WRN is essential for survival of ATL cells. Our studies suggest that targeting the WRN helicase with small inhibitors is a novel promising strategy to target HTLV-1-transformed ATL cells.

## Background

Human T-cell leukemia virus type 1 (HTLV-1) is a retrovirus that infects over 20 million people worldwide and is the etiological agent of adult T-cell leukemia/lymphoma (ATLL) [1, 2], an aggressive malignancy of mature activated T cells. HTLV-1 is associated with transformation of T lymphocytes and the development of ATLL in approximately 1–4% of infected individuals following a long latency period [3]. The diversity in clinical presentation and response to therapy of ATLL patients leads to classification into four subtypes: acute, lymphoma, chronic, and smoldering, based on organ involvement, lactate dehydrogenase (LDH), and calcium values [4]. Patients with aggressive forms of ATLL, the acute and lymphoma types, display a poor prognosis, with a median survival of approximately 1 year [5], and resistance to aggressive combined chemotherapy. Several clinical trials show that first-generation polychemotherapy containing doxorubicin (CHOP) has a limited effect on ATLL patients [6], while other approaches have yielded limited long-term benefits to ATL patients with acute or lymphoma type. In general, HTLV-1-associated disease has a poor clinical outcome, with 4-year survival rates for acute and lymphoma subtypes at 5 and 5.7%, respectively [7, 8]. The mechanism by which HTLV-1 induces T-cell transformation remains unclear; however, the viral Tax protein plays an essential role in the immortalization of human T lymphocytes [9, 10]. HTLV-1 activates an oncogenic signaling pathway, such as NF-kB and Jak/STAT [11, 12], and affects the expression of cellular miRNAs [13–16]. In addition, HTLV-1 infection leads to inactivation of several tumor suppressors and epigenetic regulators, including p16ink, Rb, p53 and p21waf, TET2, and MLL3 [17, 18]. HTLV-1-transformed cells are characterized by an increase of phospho-ATM and accumulation of ɣ-H2AX, suggesting a high level of DNA damage in those cells [19, 20]. Consistently, Tax was found to induce DNA double-strand breaks (DDSB), in an NF-kB-dependent manner, and is responsible for alteration of DNA repair machinery. Tax induces DDSB during the S-phase of the cell cycle, which are normally repaired through error-free Homologous Recombination repair (HR); however, in HTLV-1-transformed cells, the DNA damage is preferentially repaired by the error-prone non-homologous end joining (NHEJ) pathway [21, 22]. Overall, this evidence shows that Tax, by inducing DDSB and altering the DNA repair, promotes genetic instability that might be involved in the initiating events leading to transformation. Furthermore, our recent study shows that Tax-expressing cells display DNA replication issues. The replication fork progression was found to be slower and stalls more frequently in the presence of the viral protein Tax [23], suggesting that these cells might be sensitive to a DNA replication inhibitor.

Targeting DNA replication and repair machinery has been proposed as a promising strategy to combat cancer [24, 25]. Helicases are highly conserved enzymes that unwind nucleic acid duplexes during DNA replication and repair [26]. WRN mutations of the gene lead to Werner syndrome, which is characterized by genetic instability and hematological disease [27]. WRN helicases are generally highly expressed in human leukemia [28] and depletion of the gene results in mitotic catastrophe, leading to cancer cell death [29]. Interestingly, evidence shows that treatment with a WRN inhibitor (NSC 19630) significantly affects cellular proliferation of leukemia cell lines [30]. WRN helicases are involved in replication fork progression and participate in DNA double-strand break repair through homologous repair and the non-homologous end joining pathway [21]. Defects in DNA replication and DNA damage response in the HTLV-1 context lead us to hypothesize that ATL cells may be sensitive to treatment with a WRN inhibitor. Here, we demonstrate that two small WRN inhibitors, NSC 19630 and NSC 617145, induce cell cycle arrest and apoptosis in HTLV-1-transformed ATL cells.

## Methods

### Cell lines and reagent

HTLV-1-transformed cell lines (MT-4, C8166, C91PL, and 1186.94 [31–33]) and ATL-derived cell lines, IL-2-independent (ED-40515(−), TL-Om1, and ATL-25 [34, 35]), were maintained in RPMI-1640 media supplemented with penicillin, streptomycin, and 10% fetal bovine serum (FBS). ATL-derived cell lines, IL-2-dependent (LMY1, ATL-55T, ATL-43T SO4, KK1 [36–42]), were maintained in RPMI-1640 media supplemented with IL-2 (50 U/mL), penicillin, streptomycin, and 10% FBS. Peripheral blood mononuclear cells (PBMCs) were isolated from healthy donors by using Ficoll-Paque PLUS reagent (GE Healthcare Life Sciences). PBMCs were maintained in RPMI-1640 media supplemented with penicillin, streptomycin, and 20% fetal bovine serum (FBS). WRN inhibitor NSC 19630 was purchased from EMD Millipore’s Calbiochem® and NSC 617145 was purchased from Tocris Bioscience. Cells were treated with different concentrations of WRN inhibitors, and cells exposed to DMSO were used as a control, as indicated in figure legends.

### Cell cycle and proliferation assay

Cells were treated with the WRN inhibitor or DMSO as a control. After 72 h, cells were collected and washed twice with phosphate-buffered saline (PBS) and then were fixed with 80% EtOH overnight at −20 °C. The following day, cells were washed twice with PBS, incubated with RNase for 15 min at 37 °C, stained with 100 μg/mL propidium iodide (PI) for 20 min, and analyzed on an LSR II flow cytometer. Cell proliferation was measured by microscopic cell count, Cell Proliferation Kit II (XTT) (Roche), the XTT assay, according to the manufacturer’s instructions. Clonogenic assay was used to study cell proliferation. Briefly, cells were washed twice with PBS, fixed with cold Methanol (MeOH), and then stained with crystal violet dye (0.5% MeOH) for 20 min at room temperature.

### Apoptosis assay and mitochondrial membrane potential

Inhibitor-treated and control cells were collected and washed twice with PBS then stained with annexin V/propidium iodide using the Alexa Fluor® 488 Annexin V/Dead Cell Apoptosis Kit (Molecular Probes, Eugene, OR) according to the manufacturer’s instructions. Mitochondrial membrane potential (ΔΨm) was measured using the JC-1 Assay Kit (Invitrogen) according to the manufacturer’s instructions.

### Western blotting

Cell lysates were separated on SDS-PAGE followed by electroblotting to polyvinylidene difluoride membranes and probed with cyclin D1 (M-20), cyclin E (C-19), cyclin A (h432), cyclin B1 (H-20), actin (C-11), Bcl-2 (100), Mcl-1 and caspase-3 (H-60), Tax mouse monoclonal antibody (NIH AIDS Reagent Program, HTLV-I Tax Hybridoma (168B17)), and appropriate secondary antibodies purchased from Santa Cruz Biotechnology.

### Immunofluorescence

Cells were centrifuged on slides at 800 rpm for 10 min. Slides were fixed in 4% paraformaldehyde (PFA) for 15 min at room temperature and then permeabilized with 0.5% Triton X-100 for 5 min on ice for 5 min. Slides were blocked for 1 h in PBS with 0.5% gelatin and 0.25% bovine serum albumin at room temperature. For γ-H2AX and PCNA staining, slides were incubated with anti-γ-H2AX (Ser139) antibody FITC conjugated (Millipore Sigma) and PCNA antibody (DAKO, Agilent Technologies) 1/200 in PBS for 2 h, washed three times in PBS-0.2% gelatin for 10 min each time, and incubated with the appropriate Alexa Fluor secondary antibody (Molecular Probes, Invitrogen) in PBS-0.2% gelatin for 1 h at room temperature. Cells were washed three times in PBS-0.2% gelatin for 10 min each time and mounted by using DABCO mounting medium (2.5% DABCO from Sigma, 200 mM Tris-HCl pH 8.6 and 90% glycerol). Fluorescent images were captured with Nikon TE2000E epifluorescence microscope by using the ×100 objective.

### Statistical analysis

**Fig. 1:**
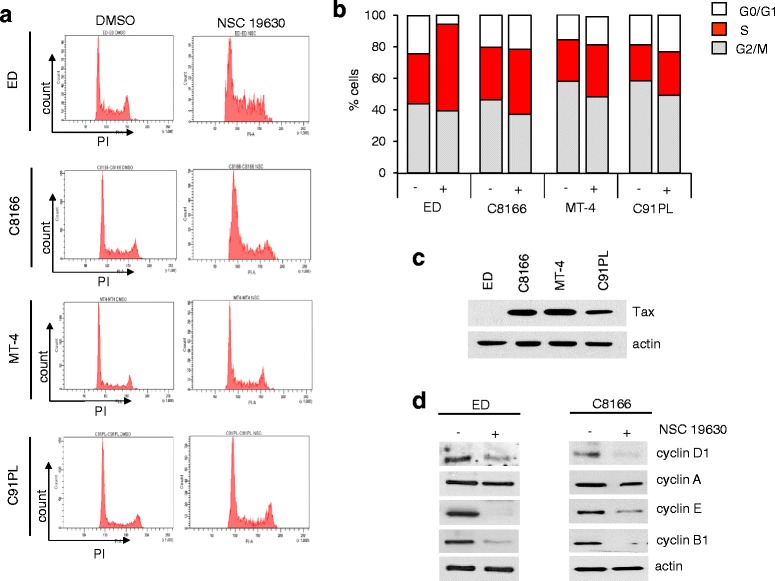
NSC 19630 inhibitor induces S-phase cell cycle arrest. **a** HTLV-1-transformed cell lines (C8166, C91PL, and MT4) and patient-derived cell lines (ED) were treated with 3 μM of NSC 19630 and DMSO vehicle has a control. After 48 h, cells were stained with propidium iodide (PI) and DNA content was analyzed by FACS to distinguish the different phases of the cell cycle (G0/G1, S, G2/M). The cell cycle analysis indicated an accumulation of the percentage of cells in S-phase, suggesting that exposure to the helicase inhibitor induced accumulation of cells in the S-phase in HTLV-1-transformed and ATL-derived cell lines. Experiment was performed multiple times in duplicate. Representative results are shown in the final figures. **b** Graphic representation of the different percentages of G0/G1-, S-, and G2/M-phase cells treated with 3 μM of NSC 19630 compared to DMSO control. **c** Western blots of Tax viral protein in ED, C8166, C91PL, and MT-4 cell lines. **d** Western blots of cyclin D1, cyclin A, cyclin E, and cyclin B1 in ED and C8166 cells following 72 h of treatment with DMSO or 3 μM of NSC 19630. Actin was used to confirm equal loading

**Fig. 2:**
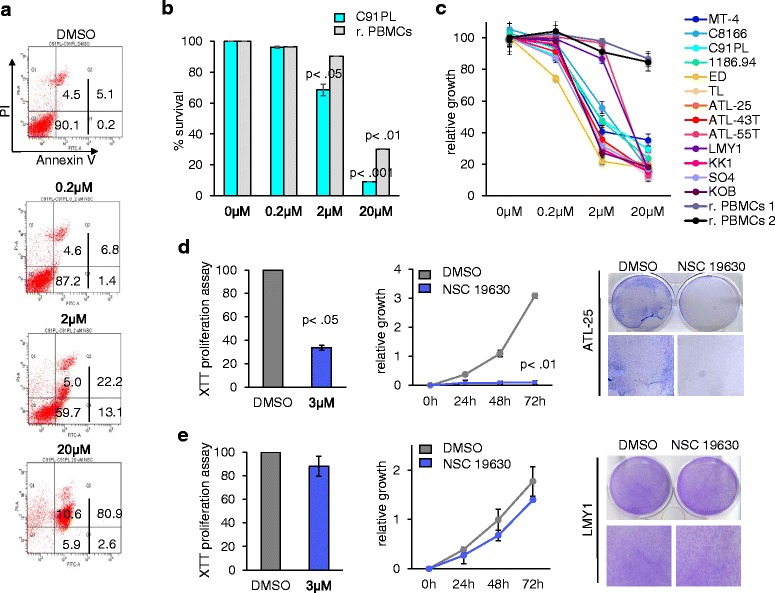
NSC 19630 inhibits cellular proliferation in patient-derived cells. **a** C91PL cells were exposed to increasing amounts of the WRN helicase inhibitor NSC 19630 (0, 0.2, 2, and 20 μM). After 72 h, cells were stained with annexin V to determine the percentage of apoptosis. The figures include the percentage of cells in the four quarters: Q1, Q2, Q3, and Q4. Q3 included the live cells that are annexin V and PI negative. Q4 included early apoptotic cells, which are annexin V positive and PI negative. Q2 included cells in late apoptosis, which are both annexin V and PI positive. Finally, Q1 included necrotic cells, which are PI positive and annexin V negative. A dose-dependent effect was noted. Experiment was performed multiple times in duplicate. Representative results are shown in the final figures. **b** Normal resting PBMCs and C91PL were exposed to increasing amounts of the WRN helicase inhibitor NSC 19630 (0, 0.2, 2, and 20 μM). After 72 h, cells were stained with annexin V and survival cells were graphed. Experiment was performed in duplicate. *P* values were calculated comparing NSC-treated cells to DMSO control by using paired and two-tailed Student’s *t* test and indicated in the figure. **c** HTLV-1-transformed (MT-4, C8166, C91PL, 1186.94) and ATL-derived (ED, TL, ATL-25, ATL-43T, ATL-55T, LMY1, KK1, SO4, KOB) cell lines and normal resting PBMCs with increasing doses of NSC 19630 (0.2, 2, and 20 μM) show inhibition of cellular growth as measured by using cell count. Experiment was performed multiple times in duplicate. Representative results are shown in the final figures. **d**, **e** Patient-derived cell lines ATL-25 and LMY1 were treated with DMSO or 3 μM of NSC 19630 for 72 h and stained with crystal violet to test the anti-proliferative property of the WRN helicase inhibitor. The results were confirmed with XTT assay and cell counts. Experiment was performed multiple times in duplicate. Representative results were shown in the final figures. *P* values were calculated by using paired and two-tailed Student’s *t* test and indicated in the figure

**Fig. 3:**
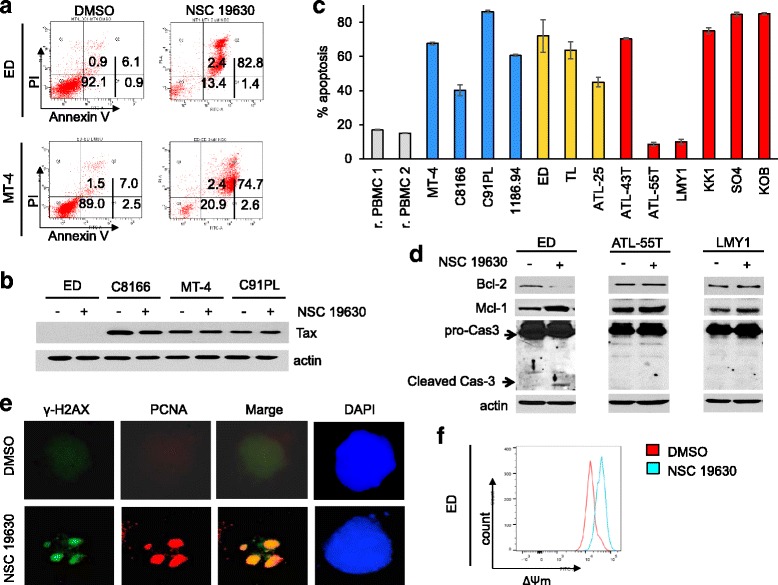
NSC 19630 induces apoptosis in HTLV-1-transformed and patient-derived cells. **a** ED and MT-4 cells were exposed to WRN helicase inhibitor NSC 19630 (3 μM) or DMSO. After 72 h, cells were stained with annexin V. The figures include the percentage of cells in the four quarters: Q1, Q2, Q3, and Q4. Q3 included the live cells that are annexin V and PI negative. Q4 included early apoptotic cells, which are annexin V positive and PI negative. Q2 included cells in late apoptosis, which are both annexin V and PI positive. Finally, Q1 included necrotic cells, which are PI positive and annexin V negative. **b** Western blots of Tax viral protein in ED, C8166, C91PL, and MT-4 cells exposed to NSC 19630 compared to DMSO-treated controls. **c** HTLV-1-transformed cell lines (MT-4, C8166, C91PL, and 1186.94) and patient-derived cell lines (ED, TL, ATL-25, ATL-43T, ATL-55T, LMY1, KK1, SO4, and KOB) were treated for 72 h with 3 μM of NSC 19630. Cells were stained with annexin V to measure the apoptotic effect of the WRN helicase inhibitor. The percentage of apoptosis and necrosis was graphed. HTLV-1-transformed, ATL-derived IL-2-dependent cell lines and IL-2-independent cell lines are represented in *blue*, *yellow*, and *red*, respectively. Tax viral protein is expressed in MT-4, C8166, C91PL, 1186.94, and ATL-25 cell lines [23]. Experiments were performed multiple times in duplicate. Representative results are shown in the final figures. **d** Western blot of Caspase-3 and apoptotic markers Bcl-2 and Mcl-1 was performed in ED, ATL-55T, and LMY1 cells exposed to DMSO or 3 μM of NSC 19630. Our analysis shows the activation of caspase-3 after treatment with the WRN helicase inhibitor. **e** Distraction of mitochondrial transmembrane potential in ED cells treated with NSC 19630 compared to DMSO control. **f** Immunofluorescence of ɣ-H2AX and PCNA in ED cells exposed for 72 h with 3 μM of NSC 19630 compared to DMSO control. Fluorescent images were captured by using the ×100 objective

**Fig. 4:**
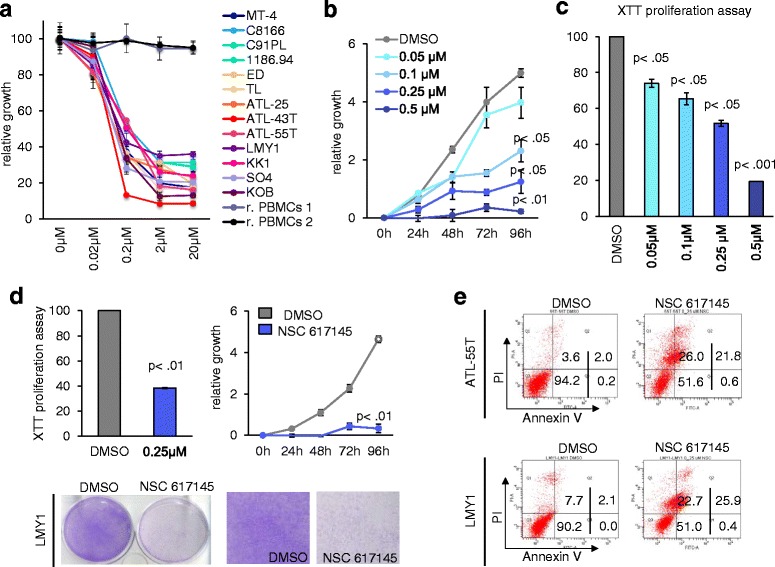
ATL-55T and LMY1 cell lines are sensitive to NSC 617145. **a** HTLV-1-transformed (MT-4, C8166, C91PL, 1186.94) and ATL-derived (ED, TL, ATL-25, ATL-43T, ATL-55T, LMY1, KK1, SO4, KOB) cell lines and normal resting PBMCs were treated with increasing doses of NSC 617145 (0.02, 0.2, 2, and 20 μM). Inhibition of cellular growth was measured by using cell count. Experiment was performed multiple times in duplicate. Representative results are shown in the final figures. **b** Patient-derived cell line, ATL-55T, was exposed to increasing amounts of NSC 617145 for 4 days, and the anti-proliferative effect was evaluated by cell count and XTT assay. Representative results are shown in the final figures. *P* values were calculated using paired two-sided Student’s *t* test. **c** LMY1-ATL-derived cells were treated with 0.25 μM of NSC 617145 for 96 h and stained with crystal violet. Cell count and XTT assay were used to measure the cellular growth of NSC 617145-treated cells compared to DMSO control cells. Experiment was performed multiple times in duplicate. Representative results are shown in the final figures. *P* values were calculated by using paired and two-tailed Student’s *t* test and indicated in the figure. **d** ATL-55T and LMY1 cells were stained with annexin V to measure the apoptotic effect of the WRN helicase inhibitor NSC 617145. Cells were treated for 92 h with 0.25 μM of NSC 617145. **e** The figures include the percentage of cells in the four quarters: Q1, Q2, Q3, and Q4. Q3 included the live cells that are annexin V and PI negative. Q4 included early apoptotic cells, which are annexin V positive and PI negative. Q2 included cells in late apoptosis, which are both annexin V and PI positive. Finally, Q1 included necrotic cells, which are PI positive and annexin V negative. Experiment was performed multiple times in duplicate. Representative results are shown in the final figures

Experiments in Figs. 1, 2, 3, and 4 were performed multiple times in duplicate. Representative results were shown in the final figures. *P* values were calculated by using paired and two-tailed Student’s *t* test. *P* values are reported in the figures and in the legends.

## Results

### NSC 19630 inhibitor induces S-phase cell cycle arrest

HTLV-1-derived cell lines and Tax-expressing cells display impaired DNA replication and repair, leading us to hypothesize that these cells may be sensitive to treatment with a small helicase inhibitor. In order to determine if the small inhibitor NSC 19630 affects cellular proliferation, we exposed in vitro HTLV-1-transformed cell lines (MT4, C8166, and C91PL) and patient-derived ATLL cell lines (ED) to 3 μM of NSC 19630 or DMSO control for 48 h. Cells were stained with propidium iodide and DNA content was analyzed by FACS. Consistent with the fact that WRN helicases are required to unwind double-stranded DNA to single-stranded DNA during DNA replication [43], NSC 19630 treatment showed significant accumulation of cells in the S-phase when compared with DMSO-exposed cells (Fig. 1a, b). Previous studies demonstrated that cells expressing a WRN-specific shRNA displayed a reduction in cellular growth [44]. In fact, WRN-depleted human fibroblasts show a marked delay in completing the cell cycle by spending more time in late S- and/or G2-phases of the cell cycle [45]. Consistent with these observations, perturbation of cell cycle progression was noted in HTLV-1-transformed and ATL-derived cell lines (Fig. 1a, b). We included Western blot of the Tax viral protein in cellular lysates derived from MT4, C8166, C91PL, and ED (Fig. 1c). As previously reported, our analysis identified ED as Tax-negative and MT4, C8166, and C91PL as Tax-positive cell lines. [23, 46]. Our analysis shows that NSC 19630 induces perturbation of cell cycle progression in both Tax-negative and Tax-positive cells.

The expression of cell cycle progression regulatory proteins was studied by Western blot in ED cells exposed to 3 μM of WRN inhibitor for 72 h. We compared the protein level of cyclins D1, E, A, and B1 in ED cells treated with NSC 19630 versus DMSO-treated controls (Fig. 1d). Cyclin E has a critical role in the control of the G1- and S-phase transitions and in the initiation of DNA replication [47]. Cyclin D1 levels vary during the cell cycle, with an elevated level of cyclin D1 maintained through G1-phase and required for the initiation of S-phase, while levels are reduced to allow DNA synthesis in S-phase [48]. However, increased levels of cyclin D1 are required to exit S-phase [48]. Similarly, cyclin A is accumulated during S-phase and is degraded before metaphase, while cyclin B1 is accumulated during the G2/M-phase [49]. Treatment of ATL cells with the WRN inhibitor NSC 19630 was associated with a decrease in the expression of cyclin D1, which may prevent treated cells from S-phase exit and result in accumulation of cells in S-phase (Fig. 1d). While there was no significant change in cyclin A expression in NSC 19630-treated cells, expression of cyclin E and cyclin B1 was significantly reduced (Fig. 1d). We believe that a relative decreased population in G2/M as a result of S-phase arrest accounts for the decrease in cyclin B1.

In addition, we decided to include Western blot of cyclins D1, E, A, and B1 in Tax-expressing cell line lysate C8166 extracted from cells exposed to NSC 19630 compared to DMSO-treated controls. Our analysis shows a reduction of cyclins E, D1, and B1 in Tax-positive cells (Fig. 1d), suggesting that cells arrested during the S-phase of the cell cycle.

Overall, our data revealed a profound alteration of the cyclin expression profile in ATL cells exposed to the WRN helicase inhibitor consistent with S-phase arrest.

### NSC 19630 inhibits cellular proliferation and induces apoptosis in HTLV-1-transformed and patient-derived cells

Recent evidence shows that treatment with the WRN inhibitor NSC 19630 significantly affects the cellular growth of different leukemia cell lines [30]. We next exposed HTLV-1-transformed C91PL to increasing logarithmic doses (0.2, 2, and 20 μM) or DMSO vehicle as a control (Fig. 2a, b). Induction of cell death was measured by using annexin V/PI staining. Apoptotic cells were scored as annexin V+, necrotic dead cells as PI+, versus live cells, which were annexin V-/PI-. We calculated the IC50 by using logarithmic transformation and we compared it to normal PBMCs isolated from healthy donors. Resting PBMCs were treated with the same concentrations of NSC 19630, 0.2, 2, and 20 μM, and induction of apoptosis by using annexin V/PI staining was measured (Fig. 2b). The IC50 in normal cells is higher compared to the HTLV-1-transformed cell line, C91PL (9.28 ± 0.23 and 2.76 ± 0.29, respectively), showing that resting PBMCs isolated from a healthy donor are less sensitive to the drug.

We expanded our analysis by testing the HTLV-1-transformed (MT-4, C8166, C91PL, 1186.94) and ATL-derived (ED, TL, ATL-25, ATL-43T, ATL-55T, LMY1, KK1, SO4, and KOB) cell lines with increasing doses of NSC 19630, 0.2, 2, and 20 μM. Inhibition of cellular growth was measured by using cell count and reported in Fig. 2c. We calculated the IC50 for every cell line by using logarithmic transformation, and the values are indicated in Table 1. Interestingly, LMY1 and ATL-55T displayed a limited reduction of cellular growth when cells were treated with 2 μM of WRN inhibitor (Fig. 3c). Consistently, the IC50 was found to be higher in those lines compared to the other cell lines tested in our study, suggesting that LMY1 and ATL-55T are less sensitive to the drug (Table 1). We included normal PBMCs isolated from healthy donors in our analysis as a negative control. As expected, limited inhibition of proliferation was noted in resting PBMCs treated with WRN inhibitor (Fig. 2c) even at high concentrations.

**Table 1:** Estimated IC50 of NSC 19630 in HTLV-1-transformed and patient-derived cells

HTLV-1-transformed cell lines	IC50 (μM)
MT-4	1.99 ± 0.065
C8166	2.84 ± 0.19
C91PL	2.76 ± 0.28
1186.94	2.23 ± 0.3
ATL-derived cell lines	IC50 (μM)
ED	0.75 ± 0.053
TL	1.73 ± 0.29
ATL-25	1.79 ± 0.22
ATL-43T	1.69 ± 0.23
ATL-55T	6.1 ± 0.15
LMY1	4.35 ± 0.21
KK1	1.64 ± 0.038
SO4	1.45 ± 0.12
KOB	1.73 ± 0.086

We tested the HTLV-1-transformed, ATL-derived cell lines (MT-4, C8166, C91PL, 1186.94, ED, TL, ATL-25, ATL-43T, ATL-55T, LMY1, KK1, SO4, KOB) with increasing doses of NSC 19630 (0.2, 2, and 20 μM), and inhibition of cellular growth was measured by using cell count. We calculated the IC50 for every cell line by using logarithmic transformation and the values are reported in the table. Our analysis shows that ATL-55T and LMY1 were found to be less sensitive compared to the HTLV-1- and ATL-derived cell lines included in the study

Our analysis shows a dose-dependent inhibition of cellular proliferation, suggesting that targeting WRN activity represents a promising strategy to kill ATL cells. These results were further confirmed by clonogenic assay. Two patient-derived ATL cell lines with adherent characteristics were exposed to 3 μM of NSC 19630 or DMSO for 72 h, washed, and then stained with crystal violet. A significant reduction in the number of cells was noted in ATL-25 (Fig. 2d); however, no significant changes were observed in LMY1, suggesting that these cells are resistant to the WRN inhibitor (Fig. 2e). To confirm these results, the anti-proliferative effect of NSC 19630 was quantified by measuring cleavage of XTT to an orange formazan dye using an ELISA reader at 450 nm and confirmed by microscopic cell count. Consistently, our analysis identified LMY1 as less sensitive to NSC 19630 (Fig. 2e). In contrast, ATL-25 was sensitive to the anti-proliferative effect of the WRN inhibitor (Fig. 2d).

The lack of effective therapeutic treatment for ATL patients led us to investigate the cytotoxicity of NSC 19630. WRN inhibitor is reported to induce cell death through accumulation of DNA double-strand breaks (DDSB) [30]. A previous article published from our laboratory shows that Tax affects the DNA repair machinery, more specifically by inhibiting Homologous Recombination (HR) repair [21]. That evidence leads us to speculate that NSC 19630 might induce cell death more prominently in Tax-expressing cells. To verify our hypothesis, we tested NSC 19630’s apoptotic effect on a Tax-expressing cell line (MT-4) versus a Tax-negative cell line (ED). Evident induction of cell death was noted in both Tax-positive and Tax-negative cells (Fig. 3a). Then we investigated if the compound affects Tax expression in three Tax-positive cell lines (C8166, MT-4, and C91PL); an ED-negative cell line was included in our analysis. Tax expression was found to be unchanged in NSC-treated cells compared to a DMSO control (Fig. 3b), suggesting that Tax expression is not a marker of drug sensitivity. A possible explanation of our finding is that Tax inhibits Homologous Recombination repair in an NF-κB-dependent manner [21]; however, constitutive activation is described in ATL cells that do not express detectable Tax [50].

Our preliminary data show that a WRN helicase inhibitor induces apoptosis in vitro (Fig. 3a), so we decided to include additional cell lines (MT-4, C8166, C91PL, and 1186.94) and nine ATL patient-derived cell lines (Fig. 3c). Previous studies suggest that IL-2-dependent ATL cell lines represent a model of smoldering and chronic forms of ATL, while IL-2-independent lines may better relate to the acute form of ATL. To investigate the potential use of WRN in various stages of ATL disease, we selected ATL-IL-2-independent (ED, TL, and ATL-25) and ATL-IL-2-dependent (ATL-43T, ATL-55T, LMY1, KK1, SO4, and KOB) cell lines for study. These cells were exposed for 72 h to the WRN helicase inhibitor and analyzed by annexin V/PI staining to measure the percentage of apoptosis induced by the compound (Fig. 3c). Significant levels of apoptosis were detected in IL-2-dependent and IL-2-independent ATL cell lines (Fig. 3c). These results suggest that HTLV-1-transformed ATL cells are highly sensitive to the WRN inhibitor NSC 19630 and that patients with the chronic, smoldering, or acute form of ATL are potential candidates for this therapeutic agent.

To gain some insights into the molecular mechanisms involved in NSC 19630’s effects on ATL cells, we next investigated disruption of the mitochondrial transmembrane potential (ΔΨm) (Fig. 3f). Activation of mitochondrial pathway cell death leads to the opening of the mitochondrial permeability transition (MPT) pore. The major consequences of this event are the disruption of ΔΨm and the release of pro-apoptotic proteins. Our analyses show that treatment with the NSC 19630 inhibitor resulted in the collapse of mitochondrial transmembrane potential in ED cells (Fig. 3f). Since the loss of mitochondrial transmembrane potential is associated with the activation of the caspase pathway [51], we investigated the activation of caspase-3, an essential mediator of apoptosis activated by proteolytic cleavage. Our data indicate that cleaved caspase-3 products were readily detected in ED-treated cells when compared to DMSO-treated control cells (Fig. 3d). We then analyzed the expression of B-cell lymphoma 2 (Bcl-2) (Fig. 3d), a protein that prevents apoptosis either by sequestering caspases or by preventing the release of mitochondrial apoptogenic factors, such as cytochrome c, and an apoptosis-inducing factor, AIF, into the cytoplasm [52]. Consistent with results from annexin V/PI, the treatment with the WRN helicase inhibitor led to decreased Bcl-2 expression in ED-treated cells (Fig. 3d). Previous studies showed that an increased expression of Mcl-1 significantly inhibits progression through the S-phase of the cell cycle [53]. Consistent with our cell cycle results demonstrating that exposure to the WRN helicase inhibitor results in S-phase arrest (Fig. 1a), increased levels of Mcl-1 were detected in ED-treated cells (Fig. 3d). Moreover, we performed Western blot of caspase-3, Bcl-2, and Mcl-1 on protein lysates of ATL-55T and LMY1 treated with DMSO or NSC 19630. As expected, no significant change of expression was noted (Fig. 3d), confirming that these lines are less sensitive to the drug.

The WRN helicase stabilizes and maintains the replication fork during DNA replication. Failure to stabilize the fork induces DNA double-strand breaks (DDSB); in fact, treatment with replication inhibitors induces fork collapse, leading to serious DNA damage and cell death [54]. Consistent with this concept, M. Aggarwal et al. demonstrated that a WRN inhibitor, NSC 19630, induces DDSB and accumulation of PCNA foci, which is associated with stalled replication forks [30]. In order to investigate if apoptosis is WRN-dependent in an HTLV-1 context, we dual-stained ɣ-H2AX (a specific marker of DDSB) and PCNA in cells exposed to NSC 19630 compared to DMSO-treated cells. Our analysis shows accumulation of PCNA and ɣ-H2AX foci, suggesting that the treatment induces DNA replication issues and, consequentially, DNA damage (Fig. 3e).

### NSC 19630-resistant ATL-55T and LMY1 cell lines are sensitive to NSC 617145

Data presented above suggest that NSC 19630 is a promising agent for the treatment of ATL. However, our analyses show that the apoptotic effect of the WRN helicase inhibitor was very limited in two ATL lines, namely LMY1 and ATL-55T (Fig.3c), suggesting potential resistance mechanisms that warrant further investigations. We studied the endogenous expression of WRN helicases in HTLV-1-transformed and ATL-derived cell lines. Consistent with previously published studies, our analyses show no direct correlation between levels of WRN protein expression and sensitivity to the WRN inhibitor (data not shown).

We then investigated NSC 617145, a WRN inhibitor identified as a close structural analog of NSC 19630 but with more potent inhibition of WRN helicase activity. We next exposed HTLV-1-transformed, ATL-derived cell lines (MT-4, C8166, C91PL, 1186.94, ED, TL, ATL-25, ATL-43T, ATL-55T, LMY1, KK1, SO4, KOB) and resting PBMCs isolated from healthy donors to increasing doses of NSC 617145 (0.02, 0.2, 2, and 20 μM), and inhibition of cellular growth was measured by cell count (Fig. 4a). We calculated the IC50 for every cell line by using logarithmic transformation and the values are reported in Table 2. Our analysis clearly shows that NSC 617145 was found to be more potent in inhibiting cellular growth compared to NSC 19630. In fact, ATL-55T cells were sensitive when exposed to increasing concentrations of NSC 617145, which inhibited cellular growth, as shown by cell count and XTT proliferation assay (Fig. 4b, c). Next, we exposed LMY1 cells to 0.25 μM for 4 days then stained with crystal violet. Consistently, again, a significant reduction in the number of cells was observed in LMY1, which was confirmed by cell count and XTT assay (Fig. 4d). Finally, we performed apoptosis assay and found that low concentrations of NSC 617145 induced high levels of cell death in both ATL-55T and LMY1 cell lines (Fig. 4e). Interestingly, limited inhibition of proliferation was noted in normal PBMCs (Fig. 4a). To confirm the limited effect of WRN inhibitor on normal cells, we estimated the IC50 in an HTLV-1-transformed cell line, C91PL, and in resting PBMCs based on induction of apoptosis. C91PL and normal resting PBMCs were exposed to increasing logarithmic doses (0.02, 0.2, and 2 μM) or DMSO vehicle as a control. Induction of cell death was measured by using annexin V/PI staining (data not show). Our analysis shows that the IC50 in normal cells is higher compared to the HTLV-1-transformed cell line C91PL (0.32 ± 0.013 and 0.13 ± 0.047, respectively), suggesting that NSC 617145 might be suitable for treating ATL patients.

**Table 2:** Estimated IC50 of NSC 617145 in HTLV-1-transformed and patient-derived cells

HTLV-1-transformed cell lines	IC50 (μM)
MT-4	0.19 ± 0.042
C8166	0.22 ± 0.023
C91PL	0.21 ± 0.0091
1186.94	0.22 ± 0.063
ATL-derived cell lines	IC50 (μM)
ED	0.15 ± 0.033
TL	0.17 ± 0.039
ATL-25	0.16 ± 0.007
ATL-43T	0.099 ± 0.013
ATL-55T	0.22 ± 0.0012
LMY-1	0.29 ± 0.0087
KK1	0.28 ± 0.032
SO4	0.14 ± 0.061
KOB	0.17 ± 0.031

We tested the HTLV-1-transformed, ATL-derived cell lines (MT-4, C8166, C91PL, 1186.94, ED, TL, ATL-25, ATL-43T, ATL-55T, LMY1, KK1, SO4, KOB) with increasing doses of NSC 617145 (0.02, 0.2, and 2 μM), and cell count was used to measure the inhibition of cellular growth. We calculated the IC50 for every cell line by using logarithmic transformation and the values are reported in the table. Our analysis clearly shows that NSC 617145 is more potent in inhibiting cellular proliferation compared to NSC 19630

Overall, our data suggest that HTLV-1-transformed ATL cells are very sensitive to the anti-proliferative and apoptotic effects of WRN helicase inhibitors.

## Discussion

In the absence of effective chemotherapy treatments, most patients with aggressive forms of the disease have a poor clinical outcome. Patients with the lymphoma type also have an unfavorable prognosis, with a median survival of 10.2 months, while patients with acute ATLL present a median survival of 6.2 months [55]. The projected 4-year survival rates of patients with the acute and lymphoma forms are only 1–5% [56]. DNA repair inhibitors induced cell death in different human leukemias [57, 58], and in the absence of an effective treatment for ATLL, we decided to test the cytotoxicity of a WRN helicase inhibitor. WRN helicases are involved in HR DNA repair and helicase activity is required during DNA replication. Previous studies showed that both DNA repair and DNA replication are impaired in HTLV-1-transformed cells.

In this study, we investigate small inhibitors of WRN as a potential therapeutic agent for ATLL. NSC 19630 targets WRN helicase activity but does not affect other DNA helicases (Bloom syndrome (BLM), Fanconi anemia group J (FANCJ), RECQ1, RecQ, UvrD, or DnaB) [30]. Our results demonstrate an apoptotic effect induced by the WRN helicase inhibitor in HTLV-1-transformed cells in vitro and in a majority of ATL-derived cell lines tested. Inhibition of cellular proliferation and induction of apoptosis were demonstrated with XTT proliferation assay, clonogenic assay, and annexin V staining. Consistent with previous studies, we observed an S-phase delay in ATL cells following treatment with NSC 19630. All the cyclins tested (cyclins E, B1, and D1) were found to be downregulated after the treatment, except for cyclin A, which is specific to the S-phase of the cell cycle. The effect of the WRN inhibitor was found to be dose- and time-dependent. Exposure to 3 μM of NSC 19630 shows significant caspase-dependent apoptosis in HTLV-1-transformed and patient-derived cells, both IL-2-dependent and IL-2-independent. We found a disruption of the mitochondrial potential, suggesting that the WRN inhibitor induces cell death through an intrinsic apoptotic pathway. Consistently, expression of Bcl-2 intrinsic anti-apoptotic factor was reduced in ED cells exposed to the WRN inhibitor. Our study shows that the WRN inhibitor efficiently kills HTLV-1-transformed and patient-derived cells. In addition, non-cancerous cells are resistant to the anti-proliferative and apoptotic effects of NSC 19630 [30], suggesting that NSC 19630 may represent a suitable strategy for initiation of phase I clinical trials. Our study also identified two cell lines less sensitive to NSC 19630, ATL-55T and LMY1. Nonetheless, these cells were efficiently killed by an NSC 19630 analog, NSC 617145, with more potent inhibitory effects on WRN helicase activity. In addition, a possible explanation of the different sensitivities to WRN helicase inhibitors in different ATL cells is that the compounds act on different domains of the enzyme. NSC 19630 is reported to inhibit the helicase domain and mildly reduce the ATPase and exonuclease activities of WRN. On the other hand, NSC 617145 reduces mainly the ATPase domain in a dose-dependent manner [59].

## Conclusions

The WRN inhibitors NSC 19630 and NSC 617145 efficiently kill HTLV-1-transformed and patient-derived cells, suggesting that WRN helicases represent a novel therapeutic target for ATLL patients.

## Abbreviations

ATLL: Adult T-cell leukemia/lymphoma
BLM: Bloom syndrome
DDSB: DNA double-strand breaks
FANCJ: Fanconi anemia group J
HR: Homologous Recombination repair
HTLV-1: Human T-cell leukemia virus type 1
IC50: Half maximal inhibitory concentration
LDH: Lactate dehydrogenase
MPT: Mitochondrial permeability transition
NHEJ: Error-prone non-homologous end joining
PBMCs: Peripheral blood mononuclear cells
WRN: Werner syndrome
